# 16p13.11 microduplication including NDE1 gene in autism spectrum disorder: A case report

**DOI:** 10.1192/j.eurpsy.2023.1883

**Published:** 2023-07-19

**Authors:** S. Moustakil, P. Guillaume, A. Letessier-Selvon

**Affiliations:** 1 Child and adolescent Psychiatry Department, Pyrenees Hospital Center, Pau, France

## Abstract

**Introduction:**

Autism spectrum disorder (ASD) is a neurodevelopmental disorder characterized by the deficit in communication and social interaction as well as restricted and repetitive interests and behaviors. In addition to the involvement of the environmental component in ASD, it is currently established the significant contribution of genetic factors such us the 16p13.11 duplication. We report the case of a patient carrying this anomaly in whom an ASD was diagnosed, the genetic study of the parents objectified the same anomaly in a clinically healthy father.

**Objectives:**

This case report aims to expand the clinical findings associated to this genomic abnormality and provide further knowledge of the pathogenic involvement of this duplication.

**Methods:**

Our patient, aged 4 years and 10 months, presented developmental peculiarities from birth. The difficulties became more evident with the language delay, the deficit of social interactions and the appearance of motor stereotypies as well as sensory specificities. The diagnosis of ASD was confirmed by the passation of the ADI and the ADOS.

Further genetic exploration with a CGH-Array request was performed due to a normal 46 XY karyotype. She objectified a 16p13.11 duplication comprising 25 genes including NDE1. The assessment was completed by the search for potentially associated malformations, particularly cardiac and skeletal. We continued the family investigation with genetic samples for the parents and the siblings finding the same anomaly in the father who does not present any particular phenotype.

**Results:**

The 16p13.11 duplication is associated with a variable clinical spectrum including behavioral disorders, attention deficit/ hyperactivity disorder, intellectual disability, cardiac and skeletal malformation, epilepsy as well as ASD and language delay presented by our patient. Eight annotated genes are present in this region including NDE1, the candidate gene for the neurocognitive phenotype. This microduplication can be found in the normal population, but it is increasingly detected in patients with ASD, schizophrenia or presenting cognitive disorders due to incomplete penetrance that can explain the presence of the duplication despite the absence of any disorders in the patient’s father.

**Conclusions:**

The link between the 16p13.11 duplication and ASD is increasingly recognized in the literature. The heterogeneity of its clinical expression and especially its incomplete penetrating make genetic counseling difficult. Collaboration between child psychiatrists and geneticists remains essential to detect, link their clinical evaluation and optimize care.

**Disclosure of Interest:**

None Declared

